# Analysis of sarcopenia parameters and falls in hemodialysis patients: no association found in a cross-sectional study

**DOI:** 10.1590/2175-8239-JBN-2025-0226en

**Published:** 2026-02-09

**Authors:** João Vitor Oblanca, Thaís Larissa Reichert, César Faúndez-Casanova, Kauana Borges Marchini, Fábio Torres, Mariana Ardengue, Sergio Seiji Yamada, Ademar Avelar

**Affiliations:** 1 Universidade Estadual de Maringá, Departamento de Educação Física, Programa de Pós-graduação Associado em Educação Física, Maringá, PR, Brazil.; 2 Universidad Católica del Maule, Facultad de Ciencias de La Educación, Escuela Pedagogía en Educación Física, Talca, Maule, Chile.; 3 Centro Universitário Cidade Verde, Maringá, PR, Brazil.; 4 Universidade Estadual de Maringá, Departamento de Medicina, Maringá, PR, Brazil.; 5 Universidade Cesumar, Maringá, PR, Brazil.; 6 Hospital Santa Casa de Maringá, Setor de Hemodiálise, Maringá, PR, Brazil.

**Keywords:** Kidney Failure, Chronic, Renal Dialysis, Muscle Weakness, Accidental Falls

## Abstract

**Introduction:**

Sarcopenia has been associated with an increased risk of falls in diverse populations. Patients with end-stage renal disease (ESRD) undergoing hemodialysis (HD) have an increased prevalence of muscle weakness and wasting. The aim of this study was to investigate the association between parameters of sarcopenia and a history of falls in ESRD patients on HD.

**Methods:**

A cross-sectional study was utilized to assess 111 participants with ESRD on HD (54 ± 15.6 years; 59.5% men). Sarcopenia was defined by low muscle strength (handgrip dynamometry) and low muscle mass (bioelectrical impedance). History of falls was self-reported. Bivariate analyses were performed, and a multivariate logistic regression model was used to assess the association between sarcopenia and falls while adjusting for confounders.

**Results:**

In the multivariate analysis, sarcopenia was not independently associated with a history of falls (OR = 1.73; *p* = 0.40). However, advanced age (OR = 1.04 per year; *p* = 0.03) and a history of stroke (OR = 6.07; *p* = 0.05) were identified as significant independent predictors of falls.

**Conclusion:**

History of falls was not independently associated with muscle strength or mass in ESRD patients on HD. Future longitudinal studies are needed to investigate other factors associated with this outcome.

## Introduction

Around 850 million people worldwide live with some form of chronic kidney disease (CKD), which is characterized by the presence of one or more markers of kidney damage and by a decreased glomerular filtration rate (GFR) (<60 ml/min/1.73 m^2^) persisting for a minimum of three months^1^. CKD stages are categorized based on this parameter. A GFR below 15 ml/min/1.73 m^2^ signifies end-stage renal disease (ESRD), requiring renal replacement therapy (RRT)^1^. Most patients with ESRD in Brazil undergo hemodialysis (HD) as their RRT of choice, with over 150,000 people in treatment in 2022^2^. CKD can be associated with complications such as hypertension, cardiovascular disease (CVD), bone mineral disorder, metabolic acidosis, and uremic symptoms, among others^3^. Common complications associated with HD treatment are intradialytic hypotension and muscle cramps, which can predispose patients to experiencing a fall^4^.

Falls occur when a person unintentionally comes to rest on the floor, the ground, or any other lower level^5^. In a study of Brazilian HD patients, it was found that 37.4% had fallen at least once in the previous year^6^. Older CKD patients fall more often than younger patients^7^. Falls increase the risk of hip and nonvertebral fractures in HD patients^8^. The use of walking aids and the presence of cardiovascular (CV) or cerebrovascular conditions increase the risk of falling in this population^9^. The post-dialysis period, the number and type of medications used, increased postural sway, and fear of falling are known risk factors for falling in this population^10,11,12,13,14,15^. Indeed, measures of poor physical function, such as reduced muscle strength, are strongly associated with falls in ESRD patients on HD^16^.

Sarcopenia, a generalized and progressive skeletal muscle disorder characterized by the accelerated loss of muscle mass and function^17^, is a key underlying factor contributing to the risk of falls in patients with ESRD on HD. The primary evidence for this is that measures of low physical function, specifically reduced muscle strength (a diagnostic parameter of sarcopenia), are strongly associated with falls in this population^16^. The prevalence of sarcopenia in the general population ranges from 5% to 10%, while studies in the HD population estimate that between 9.8% and 28.5% of patients present with the disorder, depending on the criteria used for the definition^18,19,20^. A diagnosis of sarcopenia is associated with an increased risk of mortality and CV events in CKD patients and in those undergoing HD^21,22,23^. Slow gait speed and low handgrip strength are independent predictors of fatal and non-fatal CV events in HD patients^24,25^.

Besides increasing the predisposition to falls, sarcopenia increases the overall risk of mortality, especially in community-dwelling older people^26,27,28^. The physiopathological changes associated with ESRD and HD treatment predispose this population to falls; however, the studies conducted to date have had small sample sizes and included mostly elderly patients^14,16,29^. Due to the increased risk of mortality and the predicted increase in the prevalence of CKD patients in the coming years, the impact of sarcopenia and falls in HD patients warrants further study^1^.

Given the multifactorial nature of sarcopenia in HD patients, involving nutritional deficits, inflammation, hormonal changes, and reduced physical activity, its identification and management require a collaborative approach^3,24,25^. Therefore, screening these patients by a multidisciplinary team for signs of sarcopenia is important for successful care^30^. This comprehensive assessment should include the evaluation of physical function parameters, such as gait speed and step length, as these can help identify patients at higher risk of adverse events^29,31^. Identifying patients with a fear of falling and creating safe care environments are further crucial steps to minimize fall risk^32^. Ultimately, implementing these proactive screening and prevention strategies can lead to an improved quality of life and the maintenance of functional independence during treatment^33^. Therefore, the aim of this study was to investigate the association between low muscle strength, low muscle mass, and a diagnosis of sarcopenia with falls in ESRD patients on HD. The hypothesis is that those participants who present with low muscle strength, low muscle mass, and a sarcopenia diagnosis would report more falls in the previous year.

## Methods

### Study Design

This was a single-center, cross-sectional, observational study. The manuscript was prepared following the STROBE (Strengthening the Reporting of Observational Studies in Epidemiology) statement to ensure comprehensive and transparent reporting^34^. The study was approved by the Research Ethics Committee of the *Universidade Estadual de Maringá* under protocol number 6.004.620 and was conducted in accordance with the Declaration of Helsinki. The project was also submitted for review and approval by the ethics committee of the hospital responsible for the HD center, with authorization granted for the use of clinical facilities and access to the research participants’ data. All study participants provided written informed consent before enrollment.

### Setting

The study was conducted at the nephrology unit of *Hospital Santa Casa de Maringá* (HSCM), located in Maringá, state of Paraná, Brazil. Participant recruitment and data collection took place between May and December 2023.

### Participants

The population of this study was composed of patients with ESRD undergoing HD who received care at the nephrology unit of HSCM. The participants of this study were also part of the larger project “Assessment and monitoring of global health and survival of people with chronic kidney disease undergoing hemodialysis: a cohort study,” which was approved under Public Call No. 421091/2023 – CNPq. A non-probabilistic convenience sample was utilized in this study.

The inclusion criteria were age ≥ 18 years; diagnosis of chronic kidney failure; ≥ 1 month of HD treatment at HSCM; ability to comprehend the questionnaire and participate in the physical examinations; and being clinically stable (absence of hemodynamic instability, need for intensive care, and/or any acute decompensated condition). The exclusion criteria were being permanently bedridden without the ability to walk; placement in isolated treatment due to active infection; and having any limb amputation.

**Figure 1:**
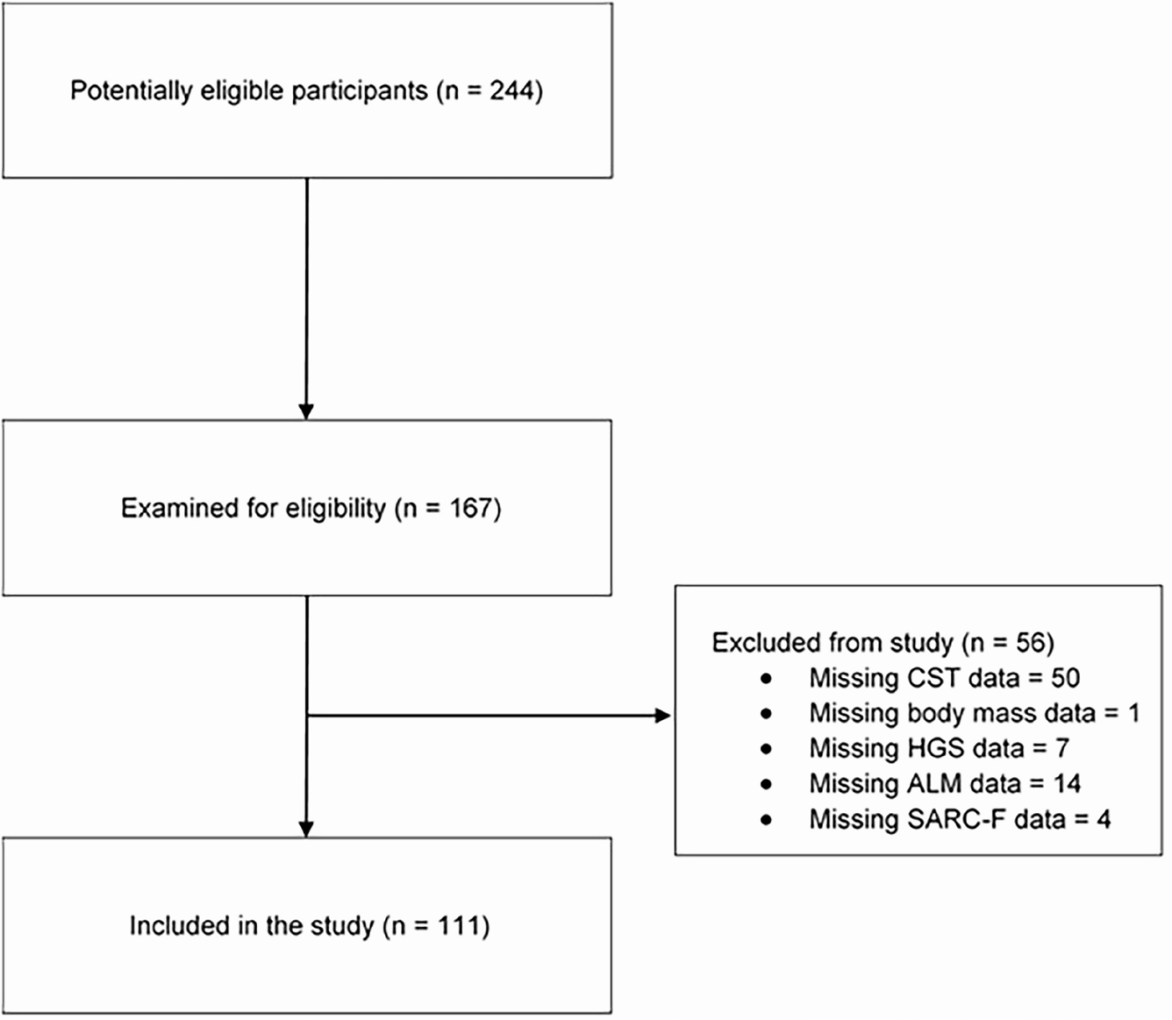
STROBE flow chart.

There were 244 patients in the HD unit of HSCM who were potentially eligible for inclusion in the study, of whom 77 did not meet the inclusion criteria. The remaining 167 participants consented to participate in the study, and their data were collected. Data from 56 of these participants were not included in the final analysis due to missing variables (missing CST [chair stand test] data = 50; missing ALM [appendicular lean mass] data = 14; missing HGS [handgrip strength] data = 7; missing SARC-F data = 4; and missing body mass data = 1). Some participants had more than one variable missing, and thus the resulting missing data number exceeds the number of participants excluded. The final sample comprised data from 111 participants (Figure 1).

To reduce potential sources of bias, such as response bias from self-reported data, all research staff were previously trained on how to administer questionnaires and conduct physical examinations.

### Variables

#### Sociodemographic and Health Data

Data were collected from all participants included in the study using a standardized questionnaire. The data collected included age, sex, race, marital status, highest level of education achieved, household income, alcohol consumption, and smoking status. Patients’ height (m), weight (kg), HD vintage, and other comorbidities were collected from their medical records. Body mass index (BMI) was calculated by dividing weight by height squared (kg/m^2^).

#### Sarcopenia

The assessment and confirmation of sarcopenia status were performed according to the definition provided in the updated version of the European Working Group on Sarcopenia in Older People (EWGSOP2)^35^. Sarcopenia status is assessed through the measurement of muscle strength, and confirmation is achieved by evaluating muscle quantity or quality.

#### Muscle Strength

Muscle strength was assessed through an HGS test using a handheld digital dynamometer (Saehan SH1001, Changwon-si, Gyeongsangnam-do, South Korea). Testing was performed in a seated position, with both feet on the ground, shoulders slightly abducted, elbows flexed to 90°, and forearms in a neutral position. HGS was assessed three times alternately in both arms, with one minute of rest between each set. Individuals were instructed to squeeze the dynamometer with maximum strength and hold for five seconds, and the highest value was recorded. The low HGS cut-off values determined by the EWGSOP2 are <27 kg and <16 kg for men and women, respectively^35^. Failure to reach these threshold values with the right hand was classified as low muscle strength.

The CST was used to assess lower limb muscle strength. Participants began the test sitting in a chair with their arms crossed over their chest, keeping their feet flat on the floor and their backs straight. They were instructed to rise to a full standing position and then sit back down again five times. The time taken to complete the test was measured using a digital chronometer. Each individual performed the test twice, and the fastest time was recorded. As per the EWGSOP2 cut-off points for the chair stand test, those who took >15 seconds to complete the test were classified as having low muscle strength^35^. Participants who presented with low muscle strength in either test were classified as having probable sarcopenia according to the EWGSOP2.

#### Muscle Mass

Lean mass was estimated through bioelectrical impedance analysis (BIA). Before the assessment, individuals were instructed to refrain from extraneous physical activity and to avoid consuming alcoholic and/or caffeinated beverages for 24 hours. The evaluation was performed using standard equipment (BIA Analyzer^TM^, The Nutritional Solutions Corporation, Harrisville, MI, USA), with patients in a supine position and electrodes placed five centimeters apart on the right hand and right foot. Resistance (Rz), reactance (Xc), and phase angle values obtained were recorded and used in the following formula to determine the participants ALM^36^:


ALM=−3.964+(0.227×RI)+(0.095×weight[kg])+(1.384×sex)+(0.064×Xc)

Where RI is the resistive index (height in centimeters squared/Rz), and sex is coded as 1 for men and 0 for women. ALM values were further corrected by their height squared (m^2^). The cut-off values for low ALM/m^2^ are <7.0 kg/m^2^ for men and <5.5 kg/m^2^ for women, as determined by the EWGSOP2 criteria^35^. Sarcopenia diagnosis was confirmed in individuals presenting with both low muscle strength and low muscle quantity, and the sample was divided into two groups: non-sarcopenic and sarcopenic.

### SARC-F

The SARC-F is a five-item questionnaire used to screen for sarcopenia risk. Individuals were asked about perceived limitations in strength, walking ability, rising from a chair, climbing stairs, and experiences with falls in the past year^35^. The fifth question (“How many times have you fallen in the past year?”) has three possible answers: (a) none; (b) one to three falls; and (c) four or more falls. Based on their answer, patients were classified into two groups: non-fallers and fallers.

### Procedures

All participant data were collected by trained and experienced professionals. All patients included in the study underwent three weekly HD sessions, spaced 48 hours apart; however, the interval between the last session of the week and the first session of the following week was 72 hours. Therefore, in an attempt to balance the participants’ health conditions, all evaluations were performed during the second and/or third HD sessions of each week. Muscle strength and mass assessments were conducted prior to the HD session, whereas sociodemographic and health data were collected during the session.

### Statistical Analysis

Statistical analyses were conducted using JASP version 0.19.3. Descriptive statistics were used to summarize the sample characteristics. Continuous and categorical variables are presented as mean ± standard deviation (SD), median and 25^th^ and 75^th^ percentiles, and percentages, respectively. Data normality was assessed using the Shapiro–Wilk test, and homogeneity was evaluated using Levene’s test. Comparisons between non-fallers and fallers were performed with Student’s *t* test, Welch’s *t* test, or the Mann-Whitney *U* test for independent variables, according to data normality and homogeneity. Cohen’s *d* was used to estimate the effect sizes (small = 0.2, medium = 0.5, and large ≥ 0.8). Associations between categorical variables were assessed with the χ^2^ test, and the effect size was estimated using Cramér’s *V* (1 df_min_ = small [0.1], moderate [0.3], and large [0.5])^37^. To assess the independent association between sarcopenia and a history of falls, a multivariate logistic regression model was used, adjusting for potential confounders (age, sex, and history of cerebrovascular disease). Results are presented as odds ratios (OR) with 95% confidence intervals (CIs). Missing data were handled using complete-case analysis. A two-sided *p*-value <0.05 was considered statistically significant.

## Results

Sociodemographic and clinical characteristics are described in Table 1. Falls in the previous year were reported by 25.2% of the sample (28/111). These individuals were classified as fallers, while the remaining participants were classified as non-fallers. Fallers were significantly older than non-fallers (60 ± 16.7 years *vs*. 51 ± 14.7 years, *t* = –2.67, *p* = 0.01). The mean difference between groups was –8.9 years, with a 95% confidence interval ranging from –15.5 to –2.3 years and a medium-to-large effect size (Cohen’s *d* = 0.58). In the unadjusted analysis, those with a previous clinical history of stroke were 1.9 times more likely to report a fall in the previous year (14.3% vs. 2.4%, χ2 = 5.78, p = 0.02). This association remained robust in the multivariate analysis. Individuals who reported a fall showed a trend toward lower ALM/m^2^ values (6.6 kg/m^2^ [6.3 – 7.1] *vs*. 7.1 kg/m^2^ [6.3 - 7.7]), although this difference did not reach statistical significance (*p* = 0.052). Among participants who experienced a fall, 46.4% had probable sarcopenia and 17.9% had confirmed sarcopenia status; these values were not significantly different from those observed among non-fallers. There were no significant differences between groups for other sociodemographic or clinical characteristics.

**Table 1:** Sociodemographic and clinical characteristics of the sample

	Total (n = 111)	No Falls (n = 83)	Falls (n = 28)	*p*
Age (years) (M ± SD)	54 ± 15.6	51 ± 14.7	60 ± 16.7	0.01^*^
Sex (%)				
*Male*	59.5	57.8	64.3	0.547
*Female*	40.5	42.2	35.7	
Height (m) (M ± SD)	1.66 ± 0.09	1.66 ± 0.09	1.66 ± 0.09	0.85
Body mass (kg) (M ± SD)	71.5 ± 15.6	72.7 ± 16.8	68.2 ± 11.1	0.12
BMI (kg/m^2^) (Median[(P25 – P75])	24.6 (22.5 - 28.2)	25.5 (22.5 - 28.7)	23.8 (22.4 - 25.8)	0.19
HD vintage (months) (Median [P25 – P75])	30.0 (12.0 - 57.5)	32.0 (16.0 - 60.0)	19.5 (10.8 - 50.5)	0.19
HGS (kg) (Median [P25 – P75])	26.9 (19.7 - 35.6)	26.9 (20.8 - 36.6)	26.4 (18.5 - 35.1)	0.57
CST (sec) (Median [P25 – P75])	11.8 (9.4 - 15.2)	11.7 (9.3 - 15.0)	12.6 (11.0 - 15.8)	0.34
ALM/m^2^ (kg/m^2^) (Median [P25 – P75])	7.0 (6.3 - 7.6)	7.1 (6.3 - 7.7)	6.6 (6.3 - 7.1)	0.05
Marital status (%)				
*Single*	19.8	21.7	14.3	0.30
*Married*	56.8	55.4	60.7	
*Cohabiting*	6.3	8.4	0.0	
*Divorced*	13.5	10.8	21.4	
*Widowed*	2.7	2.4	3.6	
Education level (%)				
*Incomplete primary education*	39.6	36.1	50.0	0.55
*Complete primary education*	13.5	14.5	10.7	
*High school diploma*	28.8	31.3	21.4	
*Bachelor’s degree*	12.6	12.0	14.3	
*Postgraduate diploma*	3.6	4.8	0.0	
*Master’s degree*	1.8	1.2	3.6	
Household income (%)				
*Up to 1 minimum wage*	15.3	15.7	14.3	0.13
*From 1 to 2 minimum wages*	28.8	25.3	39.3	
*From 2 to 6 minimum wages*	34.2	39.8	17.9	
*From 6 to 10 minimum wages*	10.8	8.4	17.9	
*More than 10 minimum wages*	8.1	9.6	3.6	
Alcohol use (%)	18.9	20.5	14.3	0.47
Tobacco use (%)	10.8	9.6	14.3	0.49
Hypertension (%)	90.1	90.4	89.3	0.87
Diabetes (%)	34.2	31.3	42.9	0.27
Dyslipidemia (%)	27.0	28.9	21.4	0.44
Heart failure (%)	9.9	7.2	17.9	0.10
Cancer (%)	2.7	2.4	3.6	0.74
Stroke (%)	5.4	2.4	14.3	0.02*
Probable Sarcopenia (%)	43.2	42.2	46.4	0.69
Confirmed Sarcopenia (%)	12.6	10.8	17.9	0.33

Abbreviations – M = mean; SD = standard deviation; BMI = body mass index; P25 = 25th percentile; P75 = 75th percentile; HD = hemodialysis; HGS = hand grip strength; CST = chair stand test; ALM/m^2^ = appendicular lean mass divided by height squared.

Notes – Student’s *t* test used for age and height; Welch’s *t* test used for body mass; Mann-Whitney *U* test used for BMI, HD vintage, HGS, CST, and ALM/m^2^; Chi-squared test used for sex, marital status, education level, household income, alcohol use, tobacco use, hypertension, diabetes, dyslipidemia, heart failure, cancer, and stroke, probable sarcopenia, and confirmed sarcopenia.

*p < 0.05.

Associations between the presence of falls in the previous year and sarcopenia-related parameters are shown in Table 2. A positive association was observed between falls reported in the past year and muscle mass status (χ^2^ = 1.13); however, this association did not reach statistical significance (*p* = 0.29) and had a small effect size (*V* = 0.10). There were no other significant associations found between the presence of falls and other sarcopenia parameters.

**Table 2:** Association between the presence of falls in the past year and parameters of sarcope

	No falls	Falls	χ^2^	*p*	*V*
HGS Classification					
Low muscle strength	23	9	0.20	0.65	0.04
Normal muscle strength	60	19			
CST Classification					
Low muscle strength	21	8	0.12	0.73	0.03
Normal muscle strength	62	20						
ALM/m^2^ Classification					
Low muscle mass	21	10	1.13	0.29	0.10
Normal muscle mass	62	18						
Probable Sarcopenia					
Present	35	13	0.16	0.69	0.04
Absent	48	15			
Confirmed Sarcopenia					
Present	9	5	0.93	0.33	0.09
Absent	74	23						

Abbreviations – χ^2^ = chi-squared test; *p* < 0.05; *V* = Cramér’s *V*; HGS = hand grip strength; CST = chair stand test; ALM/m^2^ = appendicular lean mass divided by height squared.

In the multivariate logistic regression analysis, after adjusting for covariates, sarcopenia was not identified as an independent predictor of a history of falls (OR = 1.73; *p* = 0.40). However, age (OR = 1.04 per year of increase; *p* = 0.03) and a history of stroke (OR = 6.07; *p* = 0.05) remained significant risk factors associated with falls (Table 3).

**Table 3:** Multivariate logistc regression for predictors of fall history

Variable	OR	95% CI	*p*
Sarcopenia (Present vs. Absent)	1.73	0.48 – 6.26	0.40
Age (per year of increase)	1.04	1.00 – 1.07	0.03^*^
Sex (Female vs. Male)	0.86	0.33 – 2.28	0.77
Stroke (Yes vs. No)	6.07	1.00 – 36.78	0.05

Abbreviations – OR = Odds ratio; CI = Confidence interval.

*p <0.05.

## Discussion

This study aimed to investigate the association between low muscle strength, low muscle mass, and a diagnosis of sarcopenia with falls in CKD patients on HD. It was observed that 25.2% of the sample reported at least one fall in the previous year. These participants who experienced falls were also found to be significantly older and more likely to have a previous medical history of stroke than those who did not report falls. A small association was identified between muscle mass status and a history of falls, though it was not statistically significant.

A central finding of this study was that, after multivariate adjustment, sarcopenia was not an independent predictor of falls, whereas advanced age and a history of cerebrovascular disease emerged as the most robust risk factors. This finding is consistent with other large-scale studies in dialysis and post-stroke populations^32,38,39^. The strong association with advanced age aligns with extensive literature identifying it as a primary risk factor for falls across various populations, including those undergoing HD^6,38,39,40^. In HD patients, the physiological impact of aging may be compounded by factors such as an accelerated aging process, a higher burden of comorbidities, including diabetes and CVD, and increased susceptibility to sensory decline and polypharmacy, all of which contribute to impaired postural control^3,10,11,12,13,14,15^. These findings suggest that, in the complex HD population, the impact of muscle weakness may be overshadowed by more dominant factors, such as deficits in motor control and balance associated with aging and prior neurological injury, which are known to be prevalent in these patients^32^.

The strong association with a history of stroke, which increased the odds of falls six-fold, highlights the critical importance of assessing neurological history in fall risk screening within dialysis units. Clinically, this finding is relevant, as it suggests that a simple chart review may serve as a powerful first-line screening tool. Our results imply that HD patients aged over 60 years or those with any history of stroke should be immediately triaged for a comprehensive fall assessment by a physiotherapist, regardless of sarcopenia status. This finding aligns with robust evidence identifying stroke as a major independent predictor of falls in both the general elderly population and in patients with CKD^32,38,39^. Furthermore, the lack of association with sarcopenia may have been influenced by unmeasured confounders, such as polypharmacy, physical activity levels, or objective measures of balance, which may play a more significant role than muscle parameters alone in determining fall risk in this population.

The prevalence of falls observed in the present study (25.2%) is similar to that reported by Ishii and colleagues^9^ (21.2%), whose study explored factors related to falls among older ESRD patients in the HD unit (M = 68.7 years). This prevalence underscores the significant burden of falls even within a relatively younger cohort of Brazilian HD patients, highlighting the need for routine screening in this population. The similarity to older cohorts is clinically relevant, as it suggests that the high-risk nature of the HD process itself may render even younger patients highly vulnerable to falls, reinforcing the need for fall screening among all adult HD patients, not only the elderly. In that study, the authors reported the number of falls in the previous year listed in 629 patients’ medical records. They also reported that fallers had a statistically higher chance of having a history of stroke (29.3% in fallers *vs*. 15.5% in non-fallers, *p* = 0.001), similar to what was found in the present study (14.3% in fallers *vs*. 2.4% of non-fallers, *p* = 0.02). Other studies have reported a higher prevalence of falls than that observed in the present study.

Carvalho and Dini^6^ reported that 37.4% of 131 ESRD patients on HD experienced at least one fall in the previous year. Patients included in their study were similar in age to those in the present study (M = 56.1 years) and reported fewer cases of hypertension in their cohort (66.3% among non-fallers and 33.6% among fallers) compared with our sample (90.4% among non-fallers and 89.3% among fallers). Zanotto et al.^41^ found that 37.7% of 69 participants in their cohort prospectively reported at least one fall over a 12-month period. Individuals included in that study were older than those in the present study (M = 61.7 years); however, the authors surprisingly reported that participants who fell were younger (M = 58.3 years).

Shirai et al.^16^ also reported a higher prevalence of falls among those included in their cohort (47.7% of 65 participants). The individuals with a history of falls were also found to be older (non-fallers = 71.0 years *vs*. fallers = 76.0 years, *p* = 0.55), though unlike our study, no significant difference was observed between groups. The authors also compared muscle mass, strength, and physical function data and its impact on fall frequency between the groups. Similar to the findings of the present study, muscle mass was not found to impact this outcome significantly.

Unlike the results of this study, Matsumoto et al.^42^found that, after adjusting for multiple risk factors, the hazard ratio for sarcopenia was more than fivefold higher among those who reported falling in the previous year. This result suggests that the use of the fifth question of the SARC-F questionnaire may not be the most appropriate approach to estimate the risk of falls in populations with chronic conditions.

Building on this point, the fundamental issue is that the fifth question of the SARC-F corresponds to the outcome itself, rendering its use as a “predictor” tautological, or a form of circular reasoning. This methodological flaw, which is distinct from recall bias, likely artificially inflates the observed association between the total SARC-F score and fall risk in studies that rely on this instrument.

This may help explain the discrepancy between the present findings, which did not identify sarcopenia as an independent risk factor, and those reported in studies such as that by Matsumoto. Clinically, this finding is relevant, as it serves as a critical caution against using the total SARC-F score as a proxy or substitute for a dedicated fall risk assessment. Our results would suggest that clinicians must differentiate between the valid use of the SARC-F for sarcopenia screening and the need for a separate, non-tautological tool to estimate fall risk.

This study had several strengths. First, the use of validated measures of strength and muscle mass, with appropriate cut-off values, ensured the proper estimation of these variables. Second, the large sample size allowed for appropriate estimation of the statistical significance of associations between sarcopenia parameters and falls. Finally, the use of the tools and validated questionnaires ensured real-world applicability, given their affordability and wide availability. This study also had some limitations. First, the cross-sectional design inherently limits the possibility of causal inferences. Second, the use of a self-reported questionnaire to estimate the number of falls in the previous year is subject to recall bias, and the assessment did not differentiate between single and recurrent fallers. Third, our analysis did not account for numerous key confounding factors known to be associated with falls, such as polypharmacy, physical activity, and objective measures of balance. Fourth, although missing data were handled appropriately, there were many key variables missing from some participants. Finally, the results of this study have limited generalizability, as the use of a convenience sample from a single center may limit the applicability of our findings to the broader HD population.

## Conclusion

We conclude that no association was found between muscle strength and mass parameters with a history of falls in ESRD patients on HD, an observation that persisted even after adjusting for key confounders in a multivariate analysis. Instead, we found that fall prevalence was high (25.2%), and individuals who reported falls in the previous year were older and more likely to have a history of stroke. Furthermore, this study suggests that the use of the fifth question of the SARC-F questionnaire may not be the most appropriate approach to estimate the risk of falls. Future longitudinal studies are needed to determine the predictive value of these and other factors associated with fall risk in this population.

## Data Availability

The datasets generated and/or analyzed during the current study are available from the corresponding author upon reasonable request.
