# Supplementary material for: Analysis of sarcopenia parameters and falls in hemodialysis patients: no association found in a cross-sectional study
Source: J Bras Nefrol. 2026 Feb 9;48(2):e20250226. doi: 10.1590/2175-8239-JBN-2025-0226en (PMC12904644; doi:10.1590/2175-8239-JBN-2025-0226en)
Supplement: Translation

# Translation: Análise dos parâmetros de sarcopenia e quedas em pacientes em hemodiálise: nenhuma associação encontrada em um estudo transversal

**DOI:** 10.1590/2175-8239-JBN-2025-0226pt

**Authors:** João Vitor Oblanca, Thaís Larissa Reichert, César Faúndez-Casanova, Kauana Borges Marchini, Fábio Torres, Mariana Ardengue, Sergio Seiji Yamada, Ademar Avelar

**Affiliations:** 1 Universidade Estadual de Maringá, Departamento de Educação Física, Programa de Pós-graduação Associado em Educação Física, Maringá, PR, Brasil.; 2 Universidad Católica del Maule, Facultad de Ciencias de La Educación, Escuela Pedagogía en Educación Física, Talca, Maule, Chile.; 3 Centro Universitário Cidade Verde, Maringá, PR, Brasil.; 4 Universidade Estadual de Maringá, Departamento de Medicina, Maringá, PR, Brasil.; 5 Universidade Cesumar, Maringá, PR, Brasil.; 6 Hospital Santa Casa de Maringá, Setor de Hemodiálise, Maringá, PR, Brasil.

**Keywords:** Falência Renal Crônica, Diálise Renal, Debilidade Muscular, Acidentes por Quedas

## Abstract

**Introdução:**

A sarcopenia está associada a um risco aumentado de quedas em diversas populações. Pacientes com doença renal crônica (DRC) em hemodiálise (HD) apresentam maior prevalência de fraqueza e atrofia muscular. O objetivo deste estudo foi investigar a associação entre parâmetros de sarcopenia e histórico de quedas nessa população.

**Métodos:**

Um estudo transversal foi realizado para avaliar 111 participantes com DRC em HD (54 ± 15,6 anos; 59,5% homens). A sarcopenia foi definida por baixa força muscular (dinamometria manual) e baixa massa muscular (impedância bioelétrica). O histórico de quedas foi autorreferido. Análises bivariadas foram realizadas, e um modelo de regressão logística multivariada foi utilizado para avaliar a associação entre sarcopenia e quedas, ajustado por fatores de confusão.

**Resultados:**

Na análise multivariada, a sarcopenia não foi independentemente associada ao histórico de quedas (OR = 1,73; *p* = 0,40). No entanto, a idade avançada (OR = 1,04 por ano; *p* = 0,03) e o histórico de AVC (OR = 6,07; *p* = 0,05) foram identificados como preditores independentes e significativos de quedas.

**Conclusão:**

O histórico de quedas não esteve independentemente associado à força ou à massa muscular em pacientes com DRC em HD. Estudos longitudinais futuros são necessários para investigar outros fatores associados a esse desfecho.

## Introdução

Cerca de 850 milhões de pessoas em todo o mundo vivem com alguma forma de doença renal crônica (DRC), caracterizada pela presença de um ou mais marcadores de dano renal e por uma taxa de filtração glomerular (TFG) diminuída (<60 ml/min/1,73 m^2^) por um período mínimo de três meses^1^. Os estágios da DRC são categorizados com base nesse parâmetro. Uma TFG abaixo de 15 ml/min/1,73 m^2^ caracteriza a doença renal em estágio terminal (DRCT), requerendo terapia renal substitutiva (TRS)^1^. A maioria dos pacientes com DRCT no Brasil faz hemodiálise (HD) como TRS de escolha, com mais de 150 mil pessoas em tratamento em 2022^2^. A DRC pode estar associada a complicações como hipertensão, doença cardiovascular (DCV), distúrbio mineral ósseo, acidose metabólica e sintomas urêmicos, entre outros^3^. Complicações comuns associadas ao tratamento de HD são hipotensão intradialítica e cãibras musculares, que podem predispor o paciente a sofrer uma queda^4^.

Quedas ocorrem quando uma pessoa cai involuntariamente no chão ou em qualquer outro nível inferior^5^. Em um estudo com pacientes brasileiros em HD, observou-se que 37,4% caíram pelo menos uma vez no ano anterior^6^. Pacientes idosos com DRC caem com mais frequência do que pacientes mais jovens^7^. Quedas aumentam o risco de fraturas de quadril e fraturas não vertebrais em pacientes em HD^8^. O uso de dispositivos auxiliares de marcha e a presença de condições cardiovasculares (CV) ou cerebrovasculares aumentam o risco de quedas nessa população^9^. O período pós-diálise, o número e o tipo de medicamentos usados, o aumento da oscilação postural e o medo de cair são fatores de risco conhecidos para quedas nesta população^10,11,12,13,14,15^. De fato, medidas de baixa função física, como a força muscular reduzida, estão fortemente associadas a quedas em pacientes com DRCT em HD^16^.

A sarcopenia, uma desordem musculoesquelética generalizada e progressiva caracterizada pela perda acelerada de massa e função muscular^17^, é um fator subjacente chave para o risco de quedas em pacientes com DRCT em HD. Como principal evidência, medidas de baixa função física, especificamente a força muscular reduzida (um dos parâmetros diagnósticos da sarcopenia), estão fortemente associadas a quedas nesta população^16^. A prevalência de sarcopenia na população em geral é de 5% a 10%, enquanto estudos na população em HD estimam que entre 9,8% e 28,5% dos pacientes apresentam o distúrbio, dependendo dos critérios utilizados para a sua definição^18,19,20^. O diagnóstico de sarcopenia está associado a um risco aumentado de mortalidade e de eventos CV em pacientes com DRC e naqueles submetidos à HD^21,21,23^. Marcha lenta e baixa força de preensão manual são preditores independentes de eventos CV fatais e não fatais em pacientes em HD^24,25^.

Além de aumentar a predisposição a quedas, a sarcopenia aumenta o risco geral de mortalidade, especialmente em idosos residentes na comunidade^26,27,28^. As alterações fisiopatológicas associadas à DRC em estágio terminal e ao tratamento de HD predispõem essa população a quedas, embora os estudos conduzidos apresentem amostras pequenas e incluam principalmente pacientes idosos^14,16,29^. Devido ao aumento do risco de mortalidade e à projeção de um aumento na prevalência de pacientes com DRC nos próximos anos, o impacto da sarcopenia e das quedas em pacientes em HD justifica estudos adicionais^1^.

Dada a natureza multifatorial da sarcopenia em pacientes em HD, envolvendo déficits nutricionais, inflamação, alterações hormonais e atividade física reduzida, sua identificação e manejo requerem uma abordagem colaborativa^3,24,25^. Portanto, a triagem desses pacientes por uma equipe multidisciplinar para sinais de sarcopenia é importante para o sucesso do cuidado^30^. Esta avaliação abrangente deve incluir a análise de parâmetros da função física, como velocidade da marcha e comprimento do passo, por poderem auxiliar na identificação de pacientes com maior risco de eventos adversos^29,31^. Identificar pacientes com medo de cair e criar ambientes seguros para o cuidado são passos cruciais adicionais para minimizar o risco de quedas^32^. Em última análise, a implementação dessas estratégias proativas de triagem e prevenção pode levar a uma melhoria na qualidade de vida e à manutenção da independência funcional durante o tratamento^33^. Dessa forma, o objetivo deste estudo foi investigar a associação entre baixa força muscular, baixa massa muscular e diagnóstico de sarcopenia com quedas em pacientes com DRCT em HD. A hipótese é que os participantes que apresentaram baixa força muscular, baixa massa muscular e diagnóstico de sarcopenia relataram mais quedas no ano anterior.

## Métodos

### Desenho do Estudo

Este foi um estudo observacional, transversal e unicêntrico. O manuscrito foi preparado seguindo a declaração STROBE (*Strengthening the Reporting of Observational Studies in Epidemiology*) para garantir relato abrangente e transparente^34^. A pesquisa foi aprovada pelo Comitê de Ética em Pesquisa da Universidade Estadual de Maringá, sob o número de protocolo 6.004.620, sendo conduzida de acordo com a Declaração de Helsinque. Além disso, o projeto foi submetido à revisão e aprovação do comitê de ética do hospital responsável pelo centro de HD, com autorização concedida para o uso das instalações clínicas e para o acesso aos dados dos participantes da pesquisa. Todos os participantes do estudo forneceram o consentimento informado por escrito antes da inclusão.

### Local do Estudo

O estudo foi realizado na unidade de nefrologia do Hospital Santa Casa de Maringá (HSCM), localizado em Maringá, no estado do Paraná, Brasil. O recrutamento dos participantes e a coleta de dados ocorreram entre maio e dezembro de 2023.

### Participantes

A população deste estudo foi composta por pacientes com DRCT em HD atendidos na unidade de nefrologia do HSCM. Os participantes deste estudo também fazem parte do projeto “Avaliação e monitoramento da saúde global e sobrevida de pessoas com doença renal crônica em hemodiálise: um estudo de coorte”, aprovado no Edital nº 421091/2023 — CNPq. Uma amostra não probabilística de conveniência foi utilizada neste estudo.

Os critérios de inclusão foram: idade ≥ 18 anos; diagnóstico de insuficiência renal crônica terminal; ≥ 1 mês de tratamento de HD no HSCM; capacidade de compreender os questionários e participar dos exames físicos; e estar clinicamente estável (ausência de instabilidade hemodinâmica, necessidade de terapia intensiva e/ou qualquer condição aguda descompensada). Os critérios de exclusão foram: estar permanentemente acamado, sem capacidade de deambular; estar em tratamento isolado devido à infecção ativa; ou ter qualquer amputação de membro.

Inicialmente, 244 pacientes em HD na unidade de nefrologia do HSCM eram potencialmente elegíveis para inclusão no estudo, dos quais 77 não atendiam aos critérios de inclusão. Os 167 indivíduos restantes consentiram em participar do estudo, e suas informações foram coletadas. Os dados de 56 desses participantes não foram incluídos na análise final devido a variáveis ausentes (dados de TLC [teste de levantar da cadeira] ausentes = 50; dados de MMA [massa magra apendicular] ausentes = 14; dados de FPM [força de preensão manual] ausentes = 7; dados do SARC-F ausentes = 4; e dados de massa corporal ausentes = 1). Alguns participantes apresentaram mais de uma variável ausente e, portanto, o número total de dados ausentes excedeu o número de pacientes excluídos. A amostra final foi composta por dados de 111 participantes (Figura 1).

**Figura 1:** Fluxograma STROBE.

Com o objetivo de reduzir potenciais fontes de viés, como o viés de resposta de dados autorrelatados, toda a equipe de pesquisa foi previamente treinada quanto à condução de questionários e exames físicos.

### Variáveis

#### Dados sociodemográficos e de saúde

Os dados foram coletados de todos os participantes incluídos no estudo por meio de um questionário padronizado. As informações levantadas foram: idade, sexo, raça, estado civil, maior nível de escolaridade alcançado, renda familiar, consumo de álcool e tabagismo. A altura (m), o peso (kg), o tempo de HD e outras comorbidades dos pacientes foram obtidos de prontuários médicos. O índice de massa corporal (IMC) foi calculado dividindo-se o peso pelo quadrado da altura (kg/m^2^).

#### Sarcopenia

A avaliação e a confirmação do estado de sarcopenia foi realizada de acordo com a definição da versão atualizada do Grupo de Trabalho Europeu sobre Sarcopenia em Idosos (EWGSOP2)^35^. A avaliação do estado de sarcopenia é obtida por meio da mensuração da força muscular, enquanto a confirmação é obtida pela avaliação da quantidade ou qualidade muscular.

#### Força Muscular

A força muscular foi avaliada por meio de um teste de FPM utilizando um dinamômetro digital portátil (*Saehan* SH1001, *Changwon-si*, *Gyeongsangnam-do*, Coreia do Sul). A avaliação foi realizada na posição sentada, com ambos os pés no chão, o ombro levemente abduzido, o cotovelo flexionado a 90° e o antebraço em posição neutra. A FPM foi avaliada três vezes alternadamente em ambos os braços, com um minuto de descanso entre cada série. Os participantes foram instruídos a apertar o dinamômetro com força máxima e manter a contração por cinco segundos, registrando-se o maior valor obtido. Os valores de corte para baixa FPM determinados pelo EWGSOP2 são <27 kg e <16 kg para homens e mulheres, respectivamente^35^. Aqueles que não conseguiram atingir esses valores limite com a mão direita foram classificados como tendo baixa força muscular.

O TLC foi usado para avaliar a força muscular dos membros inferiores. O teste foi iniciado com os participantes sentados em uma cadeira, com os braços cruzados sobre o peito, mantendo os pés apoiados no chão e as costas retas. Em seguida, eles foram instruídos a se levantar para uma posição totalmente em pé e, posteriormente, sentar-se novamente cinco vezes. O tempo necessário para completar o teste foi medido com um cronômetro digital. O procedimento foi repetido duas vezes e o tempo mais rápido necessário para completá-lo foi registrado. De acordo com os pontos de corte do EWGSOP2 para o teste de levantar da cadeira, aqueles que levaram mais de 15 segundos para completar o teste foram classificados como tendo baixa força muscular^35^. Indivíduos que apresentaram baixa força muscular em qualquer teste foram classificados como tendo provável sarcopenia, de acordo com o EWGSOP2.

#### Massa Muscular

A massa magra foi estimada por meio da análise de impedância bioelétrica (BIA). Os participantes foram instruídos a abster-se de atividades físicas externas e a evitar o consumo de bebidas alcoólicas e/ou com cafeína por 24 horas antes dos testes. A avaliação foi realizada com equipamento padrão (*BIA Analyzer* ^TM^, *The Nutritional Solutions Corporation*, *Harrisville*, MI, EUA) com os participantes posicionados em decúbito dorsal, e com eletrodos posicionados a cinco centímetros de distância na mão e no pé direito. Os valores de resistência (Rz), reatância (Xc) e ângulo de fase obtidos foram registrados e utilizados na seguinte fórmula para determinar a MMA dos participantes^36^:


MMA=−3,964+(0,227×IR)+(0,095×peso(kg))+(1,384×sexo)+(0,064×Xc)

Em que IR é o índice de resistência (altura em centímetros ao quadrado/Rz) e sexo corresponde a 1 para homens e 0 para mulheres. A MMA foi posteriormente corrigida pela altura ao quadrado (m^2^). Os valores de corte para MMA/m^2^ baixa são <7,0 kg/m^2^ para homens e <5,5 kg/m^2^ para mulheres, conforme determinado pelos critérios do EWGSOP2^35^. O diagnóstico de sarcopenia foi confirmado nos participantes que apresentaram baixa força muscular e baixa quantidade muscular, sendo a amostra dividida em dois grupos: não sarcopênicos e sarcopênicos.

#### SARC-F

O SARC-F é um questionário de cinco itens usado como triagem para o risco de sarcopenia. Os participantes foram questionados sobre suas limitações percebidas em termos de força, capacidade de caminhar, levantar-se de uma cadeira, subir escadas e experiências com quedas no último ano^35^. A quinta pergunta (“Quantas vezes você caiu no último ano?”) apresenta três respostas possíveis: (a) nenhuma; (b) uma a três quedas; e (c) quatro ou mais quedas. Com base nas respostas, os pacientes foram divididos em dois grupos: não-caidores e caidores.

### Procedimentos

Todos os dados dos participantes foram coletados por pesquisadores treinados e experientes. Todos os pacientes incluídos no estudo realizavam três sessões semanais de HD, com intervalo de 48 horas entre elas. Entretanto, o intervalo entre a última sessão da semana e a primeira sessão da semana seguinte era de 72 horas. Dessa forma, com o objetivo de equilibrar as condições de saúde dos participantes, todas as avaliações foram realizadas na segunda e/ou terceira sessão de HD de cada semana. As avaliações de força e massa muscular ocorreram antes da sessão de HD, enquanto dados sociodemográficos e de saúde foram coletados durante a sessão.

### Análise Estatística

As análises estatísticas foram conduzidas utilizando o software JASP, versão 0.19.3. Estatísticas descritivas foram usadas para resumir as características da amostra. Variáveis contínuas e categóricas foram apresentadas como média ± desvio padrão (DP), mediana e percentis 25 e 75 e porcentagens, respectivamente. A normalidade dos dados foi avaliada com o teste de Shapiro-Wilk, e a homogeneidade foi avaliada com o teste de Levene. A comparação entre os grupos de não-caidores e caidores foi realizada com o teste *t* de Student, teste *t* de Welch ou teste *U* de Mann-Whitney para variáveis independentes, de acordo com a normalidade e a homogeneidade. O *d* de Cohen foi utilizado para estimar os tamanhos de efeito (pequeno = 0,2; médio = 0,5; e grande ≥ 0,8). As associações entre variáveis categóricas foram avaliadas com o teste *χ^2^* e o tamanho do efeito foi estimado usando o *V* de Cramér (1 df_min_ = pequeno [0,1]; moderado [0,3]; e grande [0,5])^37^. Para avaliar a associação independente entre a sarcopenia e o histórico de quedas, foi utilizado um modelo de regressão logística multivariada para ajustar por potenciais fatores de confusão (idade, sexo e histórico de doença cerebrovascular). Os resultados foram apresentados como *odds ratios* (OR), com seus respectivos intervalos de confiança (IC) de 95%. Dados ausentes foram tratados usando análise completa de caso. Um valor de *p* bilateral <0,05 foi considerado estatisticamente significativo.

## Resultados

As características sociodemográficas e clínicas dos participantes estão descritas na Tabela 1. Quedas no ano anterior foram relatadas por 25,2% da amostra (28/111), que compuseram o grupo de caidores, enquanto os demais foram classificados como não-caidores. Os caidores eram significativamente mais velhos do que os não-caidores (60 ± 16,7 anos vs. 51 ± 14,7 anos; *t* = -2,67; *p* = 0,01). Houve uma diferença média de -8,9 anos, com um intervalo de confiança de 95% entre -15,5 a -2,3 anos entre os grupos, com um tamanho de efeito médio a grande (*d* de Cohen = 0,58). Na análise não ajustada, indivíduos com histórico clínico prévio de acidente vascular cerebral (AVC) tiveram 1,9 vezes maior probabilidade de relatar uma queda no ano anterior (14,3% vs. 2,4%; χ2 = 5,78; p = 0,02). Essa associação permaneceu robusta na análise multivariada. Aqueles que relataram uma queda mostraram tendência a apresentar menor MMA/m^2^ (6,6 kg/m^2^ [6,3-7,1] vs. 7,1 kg/m^2^ [6,3-7,7]), mas essa diferença não atingiu significância estatística (*p* = 0,052). Entre os participantes que sofreram queda, 46,4% apresentavam sarcopenia provável, enquanto 17,9% tinham *status* de sarcopenia confirmado; esses valores não foram significativamente diferentes daqueles observados entre os participantes que não caíram. Não houve diferenças significativas entre os grupos para nenhuma outra característica sociodemográfica ou clínica.

**Tabela 1:** Características sociodemográficas e clínicas da amostra

	Total (n = 111)	Não-caidores (n = 83)	Caidores (n = 28)	*p*
Idade (anos) (M ± DP)	54 ± 15,6	51 ± 14,7	60 ± 16,7	0,01^*^
Sexo (%)				
*Homens*	59,5	57,8	64,3	0,55
*Mulheres*	40,5	42,2	35,7	
Altura (m) (M ± DP)	1,66 ± 0,09	1,66 ± 0,09	1,66 ± 0,09	0,85
Massa corporal (kg) (M ± DP)	71,5 ± 15,6	72,7 ± 16,8	68,2 ± 11,1	0,12
IMC (kg/m^2^) (Mediana [P25 – P75])	24,6 (22,5 – 28,2)	25,5 (22,5 – 28,7)	23,8 (22,4 – 25,8)	0,19
Tempo em HD (meses) (Mediana [P25 – P75])	30,0 (12,0 – 57,5)	32,0 (16,0 – 60,0)	19,5 (10,8 – 50,5)	0,19
FPM (kg) (Mediana [P25 – P75])	26,9 (19,7 – 35,6)	26,9 (20,8 – 36,6)	26,4 (18,5 – 35,1)	0,57
TLC (s) (Mediana [P25 – P75])	11,8 (9,4 – 15,2)	11,7 (9,3 – 15,0)	12,6 (11,0 – 15,8)	0,34
MMA/m^2^ (kg/m^2^) (Mediana [P25 – P75])	7,0 (6,3 – 7,6)	7,1 (6,3 – 7,7)	6,6 (6,3 – 7,1)	0,05
Estado civil (%)				
*Solteiro(a)*	19,8	21,7	14,3	0,30
*Casado(a)*	56,8	55,4	60,7	
*União estável*	6,3	8,4	0,0	
*Divorciado(a)*	13,5	10,8	21,4	
*Viúvo(a)*	2,7	2,4	3,6	
Nível de escolaridade (%)				
*Ensino fundamental incompleto*	39,6	36,1	50,0	0,55
*Ensino fundamental completo*	13,5	14,5	10,7	
*Ensino médio completo*	28,8	31,3	21,4	
*Bacharelado(a)*	12,6	12,0	14,3	
*Pós-graduado*	3,6	4,8	0,0	
*Mestrado*	1,8	1,2	3,6	
Renda familiar (%)				
*Até 1 salário mínimo*	15,3	15,7	14,3	0,13
*De 1 a 2 salários mínimos*	28,8	25,3	39,3	
*De 2 a 6 salários mínimos*	34,2	39,8	17,9	
*De 6 a 10 salários mínimos*	10,8	8,4	17,9	
*Mais de 10 salários mínimos*	8,1	9,6	3,6	
Uso de álcool (%)	18,9	20,5	14,3	0,47
Uso de tabaco (%)	10,8	9,6	14,3	0,49
Hipertensão arterial (%)	90,1	90,4	89,3	0,87
Diabetes (%)	34,2	31,3	42,9	0,27
Dislipidemia (%)	27,0	28,9	21,4	0,44
Insuficiência cardíaca (%)	9,9	7,2	17,9	0,10
Câncer (%)	2,7	2,4	3,6	0,74
AVC (%)	5,4	2,4	14,3	0,02^*^
Provável sarcopenia (%)	43,2	42,2	46,4	0,69
Sarcopenia confirmada (%)	12,6	10,8	17,9	0,33

Abreviaruras – M = média; DP = desvio padrão; IMC = índice de massa corporal; P25 = 25° percentil; P75 = 75° percentil; HD = hemodiálise; FPM = força de preensão manual; TLC = teste de levantar da cadeira; MMA/m^2^ = massa magra apendicular dividida pela altura ao quadrado; AVC = acidente vascular cerebral.

Notas – Teste *t* de Student usado para idade e altura; teste *t* de Welch usado para massa corporal; teste *U* de Mann-Whitney usado para IMC, tempo de HD, FPM, TLC e MMA/m^2^; teste qui-quadrado usado para sexo, estado civil, nível de escolaridade, renda familiar, uso de álcool, uso de tabaco, hipertensão arterial, diabetes, dislipidemia, insuficiência cardíaca, câncer e AVC, sarcopenia provável e sarcopenia confirmada.

*p < 0,05.

As associações entre a presença de quedas no último ano e os parâmetros de sarcopenia são apresentadas na Tabela 2. Foi encontrada uma associação positiva entre as quedas relatadas no último ano e o estado da massa muscular (β = 1,13), embora não tenha sido alcançada significância estatística (*p* = 0,29). Essa associação teve um tamanho de efeito pequeno (*V* = 0,10). Não foram encontradas outras associações significativas entre a presença de quedas e os demais parâmetros de sarcopenia.

**Tabela 2:** Associação entre a presença de Quedas no último sno e os parâmetros de sarcopenia

	Não-caidores	Caidores	χ^2^	*p*	*V*
Classificação FPM					
Baixa força muscular	23	9	0,20	0,65	0,04
Força muscular normal	60	19						
Classificação TLC					
Baixa força muscular	21	8	0,12	0,73	0,03
Força muscular normal	62	20						
Classificação MMA/m2					
Baixa massa muscular	21	10	1,13	0,29	0,10
Massa muscular normal	62	18						
Provável sarcopenia					
Presente	35	13	0,16	0,69	0,04
Ausente	48	15						
Sarcopenia confirmada					
Presente	9	5	0,93	0,33	0,09
Ausente	74	23						

Abreviaturas – χ^2^ = teste qui-quadrado; *p* < 0,05; *V* = *V* de Cramér; FPM = força de preensão manual; TLC = teste levantar da cadeira; MMA/m^2^ = massa magra apendicular dividida pela altura ao quadrado.

Na análise de regressão logística multivariada, após ajuste para as covariáveis, a sarcopenia não se mostrou um preditor independente do histórico de quedas (OR = 1,73; *p* = 0,40). No entanto, a idade (OR = 1,04 por ano de aumento; *p* = 0,03) e o histórico de AVC (OR = 6,07; *p* = 0,05) permaneceram como fatores de risco significativamente associados às quedas (Tabela 3).

**Tabela 3:** Análise de regressão logística multivariada para preditores do histórico de quedas

Variável	OR	IC 95%	*p*
Sarcopenia (Presente vs. Ausente)	1,73	0,48 – 6,26	0,40
Idade (por ano de aumento)	1,04	1,00 – 1,07	0,03^*^
Sexo (Mulher vs. Homem)	0,86	0,33 – 2,28	0,77
AVC (Sim vs. Não)	6,07	1,00 – 36,78	0,05

Abreviaturas – OR = *Odds Ratio*; IC = Intervalo de Confiança; AVC = Acidente vascular cerebral.

*p < 0,05.

## Discussão

Este estudo teve como objetivo investigar a associação entre baixa força muscular, baixa massa muscular e diagnóstico de sarcopenia com a ocorrência de quedas em pacientes com DRC em HD. Constatamos que 25,2% dos participantes relataram ter sofrido quedas pelo menos uma vez no ano anterior. Os participantes que sofreram quedas eram significativamente mais velhos e mais propensos a apresentar histórico médico prévio de AVC do que aqueles que não relataram quedas. Houve uma associação pequena entre o estado da massa muscular e o histórico de quedas, embora essa associação não tenha sido estatisticamente significativa.

Um achado central deste estudo foi que, após ajuste multivariado, a sarcopenia não se apresentou como um preditor independente de quedas. Em contraste, a idade avançada e o histórico de doença cerebrovascular emergiram como os fatores de risco mais robustos, em consonância com outros estudos em larga escala em populações de diálise e pós-AVC^32,38,39^. A forte associação com a idade avançada está em consonância com a vasta literatura que a identifica como um fator de risco primário para quedas em diversas populações, incluindo aquelas em HD^6,38,39,40^. Em pacientes em HD, o impacto fisiológico do envelhecimento pode ser agravado por fatores como o processo de envelhecimento acelerado, a maior carga de comorbidades, como diabetes e DCV, e a maior suscetibilidade ao declínio sensorial e à polifarmácia, contribuindo para o comprometimento do controle postural^3,10,11,12,13,14,15^. Este resultado sugere que, na população complexa de pacientes em HD, o impacto da fraqueza muscular pode ser sobreposto por fatores mais dominantes, como os déficits no controle motor e no equilíbrio associados ao envelhecimento e a lesões neurológicas prévias, reconhecidamente prevalentes nesses pacientes^32^.

A forte associação com o histórico de AVC, que aumentou a chance de quedas em aproximadamente seis vezes, ressalta a importância crítica da avaliação do histórico neurológico na triagem do risco de quedas em unidades de diálise. Clinicamente, este achado é altamente relevante, ao sugerir que uma simples análise do prontuário médico é uma poderosa ferramenta de triagem de primeira linha. Nossos resultados implicam que pacientes em HD com mais de 60 anos e/ou com histórico de AVC devem ser imediatamente encaminhados para uma avaliação completa do risco de quedas por um fisioterapeuta, independentemente do estado de sarcopenia. Isso está alinhado com evidências robustas que identificam o AVC como um importante preditor independente de quedas tanto na população idosa em geral quanto em pacientes com DRC^32,38,39^. Adicionalmente, é possível que a ausência de associação com a sarcopenia tenha sido influenciada por confundidores não mensurados, como polifarmácia, nível de atividade física ou medidas objetivas de equilíbrio. Esses fatores podem desempenhar um papel mais significativo do que os parâmetros musculares, considerados isoladamente, na determinação do risco de quedas nessa população.

A prevalência de quedas encontrada em nosso estudo (25,2%) é semelhante à observada por Ishii et al.^9^ (21,2%), cujo estudo explorou os fatores relacionados a quedas que ocorreram em pacientes idosos com DRC em uma unidade de HD (M = 68,7 anos). Tal achado ressalta a carga significativa das quedas, mesmo em uma coorte relativamente mais jovem de pacientes brasileiros em HD, destacando a necessidade de rastreamento rotineiro nessa população. Essa semelhança com os pacientes idosos é clinicamente significativa, por sugerir que a natureza de alto risco do próprio processo de HD pode tornar até mesmo pacientes mais jovens altamente vulneráveis, reforçando a necessidade de rastreamento de quedas em todos os pacientes adultos em HD, e não somente nos idosos. Os autores relataram o número de quedas no ano anterior listadas nos prontuários médicos de 629 pacientes. Além disso, relataram que aqueles que sofreram quedas tinham uma chance estatisticamente maior de ter histórico de AVC (29,3% entre os que sofreram quedas vs. 15,5% entre os que não sofreram quedas; *p* = 0,001), semelhante ao que foi encontrado no presente estudo (14,3% entre os que sofreram quedas vs. 2,4% entre os que não sofreram quedas; *p* = 0,02). Outros trabalhos descreveram uma prevalência de quedas maior do que a encontrada em nosso estudo.

Carvalho e Dini^6^ observaram que 37,4% dos 131 pacientes com DRC em HD sofreram pelo menos uma queda no ano anterior. Os pacientes incluídos em seu estudo eram semelhantes em idade aos participantes de nosso estudo (M = 56,1 anos) e apresentaram menos casos de hipertensão em sua coorte (66,3% entre não-caidores e 33,6% entre caidores) em comparação com a nossa (90,4% entre não-caidores e 89,3% entre caidores). Zanotto et al.^41^ observaram que 37,7% dos 69 participantes incluídos em sua coorte prospectiva relataram ter caído pelo menos uma vez durante 12 meses. Os pacientes incluídos em sua pesquisa eram mais velhos do que os do nosso estudo (M = 61,7 anos), embora, surpreendentemente, os autores tenham relatado que os participantes que caíram eram mais jovens (M = 58,3 anos).

Shirai et al.^16^ também relataram uma maior prevalência de quedas entre os participantes de sua coorte (47,7% dos 65 participantes). Aqueles com histórico de quedas também eram mais velhos (não-caidores = 71,0 anos vs. caidores = 76,0 anos; *p* = 0,55), embora, diferentemente do nosso estudo, não tenha sido encontrada diferença significativa entre os grupos. Os autores também compararam os dados de massa muscular, força e função física e seu impacto na frequência de quedas entre os grupos. Similarmente ao que foi observado em nosso estudo, a massa muscular não impactou significativamente esse desfecho.

Em contraste com os resultados deste trabalho, Matsumoto et al.^42^ constataram que, após o ajuste para múltiplos fatores de risco, a razão de risco de sarcopenia foi mais de cinco vezes maior entre aqueles que relataram queda no ano anterior. Esse resultado sugere que a utilização da quinta pergunta do questionário SARC-F pode não ser a maneira mais adequada de estimar o risco de quedas em populações com condições crônicas.

Com base nesse ponto, a questão fundamental é que a quinta pergunta do SARC-F representa o próprio desfecho, tornando seu uso como “preditor” tautológico, ou seja, uma forma de raciocínio circular. Essa falha metodológica, distinta do viés da memória, provavelmente influencia artificialmente a associação observada entre a pontuação total do SARC-F e o risco de quedas em estudos que se baseiam nesse instrumento.

Esse aspecto pode ajudar a explicar a discrepância entre nossos achados, que não identificaram a sarcopenia como um fator de risco independente, e os resultados de estudos como o de Matsumoto. Clinicamente, esse achado é altamente relevante, ao servir como um alerta importante contra o uso da pontuação total do SARC-F como um indicador ou substituto para uma avaliação específica do risco de quedas. Nossos resultados sugerem que os clínicos devem diferenciar entre o uso válido do SARC-F para o rastreamento da sarcopenia e a necessidade de uma ferramenta distinta e não tautológica para estimar o risco de quedas.

Este estudo apresentou alguns pontos fortes. Primeiro, o uso de medidas validadas de força e massa muscular, com valores de corte apropriados, garantiu uma estimativa adequada dessas variáveis. Segundo, a ampla amostra permitiu a estimativa correta da significância estatística das associações entre os parâmetros de sarcopenia e as quedas. Finalmente, o uso de ferramentas e questionários validados garantiu a aplicabilidade no mundo real devido à sua acessibilidade e ampla disponibilidade. O presente trabalho também apresentou algumas limitações. Primeiro, o delineamento transversal limita inerentemente a possibilidade de inferências causais. Segundo, o uso de um questionário autorrelatado para estimar o número de quedas no ano anterior está sujeito ao viés de memória dos participantes, e a avaliação não diferenciou entre quedas únicas e recorrentes. Terceiro, nossa análise não considerou diversos fatores de confusão importantes, reconhecidamente associados a quedas, como a polifarmácia, o nível de atividade física e as medidas objetivas de equilíbrio. Quarto, embora os dados ausentes tenham sido tratados adequadamente, havia um grande número de variáveis-chave ausentes em alguns dos participantes. Finalmente, os resultados deste estudo apresentam generalização limitada; o uso de uma amostra de conveniência de um único centro pode limitar a aplicabilidade dos nossos achados à população mais ampla de pacientes em HD.

## Conclusão

Concluímos que não houve associação entre os parâmetros de força e massa muscular e o histórico de quedas em pacientes com DRC em HD, uma observação que se manteve mesmo após o ajuste por importantes fatores de confusão em uma análise multivariada. Em vez disso, observou-se que a prevalência de quedas foi alta (25,2%) e que os pacientes que relataram quedas no ano anterior eram mais velhos e apresentavam histórico de acidente vascular cerebral (AVC). Além disso, este estudo sugere que a utilização da quinta pergunta do questionário SARC-F pode não ser a maneira mais apropriada para estimar o risco de quedas. Estudos longitudinais futuros são necessários para determinar o valor preditivo desses e de outros fatores associados ao risco de quedas nessa população.

## Disponibilidade de Dados

Os conjuntos de dados gerados e/ou analisados durante o atual estudo estão disponíveis com o autor correspondente mediante solicitação razoável.
